# Monitoring of Organochlorine Pesticide and Polychlorinated Biphenyl Residues in Common Swifts (*Apus apus*) in the Region of Hannover, Lower Saxony, Germany

**DOI:** 10.3390/vetsci8050087

**Published:** 2021-05-16

**Authors:** Warakorn Tiyawattanaroj, Stefan Witte, Michael Fehr, Marko Legler

**Affiliations:** 1 Clinic for Small Mammals, Reptiles and Birds, University of Veterinary Medicine Hannover, Foundation, 30559 Hannover, Germany; michael.fehr@tiho-hannover.de; 2 Chemisches Labor Dr. Wirts und Partner Sachverständigen GmbH, 30559 Hannover, Germany; witte@wirts.de

**Keywords:** Apodidae, DDE, migratory bird, OCP, PCB, wild birds, common swift

## Abstract

The use of pesticides is associated with the decline of several avian species. In this study, we monitored the organochlorine contaminants in common swifts (*Apus apus*) in the years 2016 to 2018. These long-distance migrants breed in Europe and winter in Africa. Their only feeding source is aerial plankton. Pooled organ samples of 42 adult and 40 juvenile swifts were tested with the multi-residue method by gas chromatography-mass spectrometry (GC-TOF/MS). Predominantly, 4,4′-DDE, dieldrin, hexachlorobenzene (HCB), lindane and polychlorinated biphenyls (PCBs) were found in most of these common swifts. Only 4,4′-DDE (adult: 83 ± 70 μg/kg, juvenile: 17 ± 39 μg/kg) and dieldrin (adult: 2 ± 3 μg/kg, juvenile: 0.3 ± 1 μg/kg) concentrations were significantly different between adult and juvenile birds. All detected concentrations in our study were far lower than the previously recorded pesticide concentrations of common swifts in Italy and those which are known to cause toxicity and death in birds.

## 1. Introduction

The globally declining population of many avian species has various causes, including habitat loss, climatic change and environmental pollutants [1,2,3,4]. Pollutants such as persistent pesticides not only have a long-term impact on the environment but are also accumulated in the different bird species [5,6,7,8]. An illustrative example of a study on a pesticide that affected birds showed that the dichlorodiphenyltrichloroethane (DDT) metabolite, dichlorodiphenyldichloroethylene (DDE) was a cause of eggshell thinning, with a major effect on reproduction and hatching success [9,10]. For that reason, many organochlorine pesticides (OCPs), including DDT have been prohibited in most countries since the early 1970s [7,11]. A recent study of contamination in the blood of migratory birds such as kittiwakes (*Rissa tridactyla*) has shown that they can ingest food contaminated with OCPs, including DDE even though it is located in the high Arctic Ocean of Norway [12]. This indicates that these long-term residual chemicals have remained across the world. Long-distance migrants may have a higher chance of being exposed to large-scale pollution, which could be from the breeding area, the migration route and the wintering ground [2,8,13]. The World Health Organization has found that OCPs continue to be used as indoor residual spraying agents for malaria vector prevention in 80 malaria-endemic countries, particularly in Africa between 2010 to 2018, including in the Congo region [14,15]. The use of these products has become commonplace in developing regions [16]. The biggest potential for the disastrous effects of pesticides is through contamination of the hydrological system, which supports human life, the aquatic ecosystem, and related food chains [17].

The common swift (*Apus apus*) is an insectivorous migratory bird and the most frequently encountered species of the order Apodiformes, which breeds through Europe and winters in Sub-Saharan Africa, mainly in the Congo region [18,19]. Recording the migration routes and migration periods of these particular birds showed that they spend most of their time flying [20]. Their prey consists of a wide variety of insects and arachnids, which are known as aerial plankton [21]. Common swifts have suffered the largest decline among 50 species of birds found in gardens that are observed by birdwatchers from all regions in Germany. Their population declined 6.7% annually on average from 2006 to 2018 [22].

Common swifts were once used as a bioindicator in Rome, Italy, to study persistent micro-organic contaminants such as polychlorinated biphenyls (PCBs), polychlorodibenzo-p-dioxins (PCDDs), polychlorodibenzofurans (PCDFs) as well as the chlorinated pesticides DDE, DDT and hexachlorobenzene (HCB), which were analysed in the organ tissue of adult swifts. The results suggested that this species was a suitable biomonitor for evaluating air pollution [23,24].

In this study, we monitored the organochlorine pesticide and polychlorinated biphenyl residues in common swifts in the area of Hannover, Lower Saxony, Germany, and evaluated our assumption that there were significant differences in chemical levels between adult and juvenile swifts.

## 2. Materials and Methods

### 2.1. Birds and Ethical Statement

From April to September from 2016 to 2018, 42 fatally injured grounded adult and 40 juvenile common swifts were brought by locals to the Clinic for Small Mammals, Reptiles and Birds, University of Veterinary Medicine Hannover, Foundation Germany. The age of the swifts was ascertained from the typical colour, the shape of the feathers and the moulting pattern [21]. The nestlings from different development stages, which had not yet completed the growth of the primary flight feathers, were categorised as juvenile birds in this study. All birds died or were euthanised due to the poor prognosis, from both clinical examination and radiographic findings, meaning severe fractures, which could not be rehabilitated successfully according to the guideline of Haupt (2001) [25]. The birds were stored at −20 °C awaiting further investigation.

The Ethics Committee of the University of Veterinary Medicine of Hannover, Foundation approved all procedures of obtaining samples for analysis used in this study (ethic code: TVO-2018-V-18). The use of death animals complied with German animal welfare laws, guidelines, and policies; the study did not involve living animals.

### 2.2. Samples

Within 2 months of storage, the birds were then thawed at room temperature for a full necropsy. Pooled organ samples of each bird (breast muscle, fat tissue, liver, kidney, lungs and brain) were taken immediately in order to minimise the microbiological degradation and prevent the analytes loss, using sterile equipment and refrozen at −20 °C until the analysis for different chemical residues. 

Due to the insufficient organ volumes in each bird, one sample in each test consisted of pooled organ samples from two birds, which were matched to achieve the minimum volume of 4 g for analysis under the same conditions of age and the year of collection (41 paired samples from 82 birds) over 3 years.

### 2.3. Chemical Analysis

The pooled samples were analysed for 44 selected OCPs and 6 PCBs by the Chemisches Labor Dr. Wirts + Partner Sachverständigen GmbH in Hannover, after a maximum of 1-week storage. The quantification limits of the OCPs and PCBs are shown in Table 1. The analysis of residues in the sample was performed on gas chromatography with time-of-flight and mass spectrometer (GC-TOF/MS) by the multi-residue method (MRM) ASU § 64 LFBG, according to S19 of DFG pesticide commission.

### 2.4. Statistical Analysis

The statistical analysis of the results was performed with the software package IBM SPSS Statistics 26. After running Kolmogorov–Smirnov test, it was shown that the pesticide concentrations were non-parametric distribution. On this basis, the Mann–Whitney U test was chosen to compare the concentration of pesticides between the adult and juvenile common swifts. A significance level *p* ≤ 0.05 was chosen.

## 3. Results

**Figure 1:**
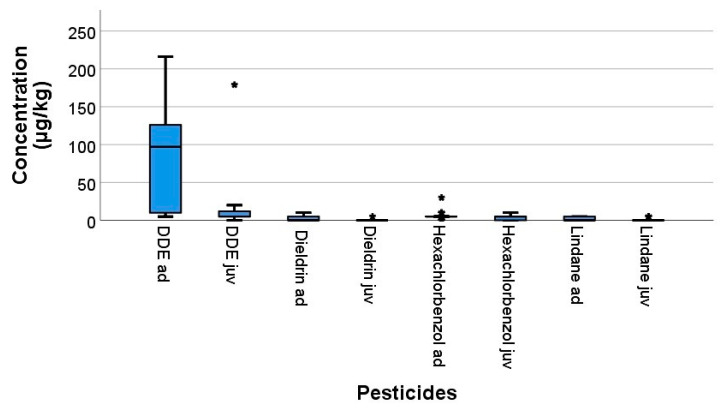
Concentrations of pesticide in organs of common swifts detected in 2016–2018 in µg/kg wet weight. The box plot shows the median (horizontal line in centre), the 25th percentile (box border at bottom) and the 75th percentile (box border at top). The upper and lower ends of the whisker show the maximum and minimum values. The single data points are outliers. The asterisks are extreme outliers.

In the pooled organ samples of the examined swifts, 4 OCPs were detected in the area of Hannover, Germany from 2016 to 2018, which include: 4,4′-DDE, dieldrin, HCB and lindane (Figure 1). The results are shown separately by year in Table 2. The 4,4′-DDE were detected in all the pooled samples of the adult birds and in 85% of the juvenile bird samples, followed by HCB (adult: 76.2%, juvenile: 65%), lindane (adult: 38% and juvenile: 15%), and dieldrin (adult: 33.3%, juvenile: 5%).

The concentrations of 4,4′-DDE (*p* = 0.002; Mann–Whitney U test) and dieldrin (*p* = 0.023; Mann–Whitney U test) in the organs of adults were significantly higher than in juvenile swifts (Table 3).

**Figure 2:**
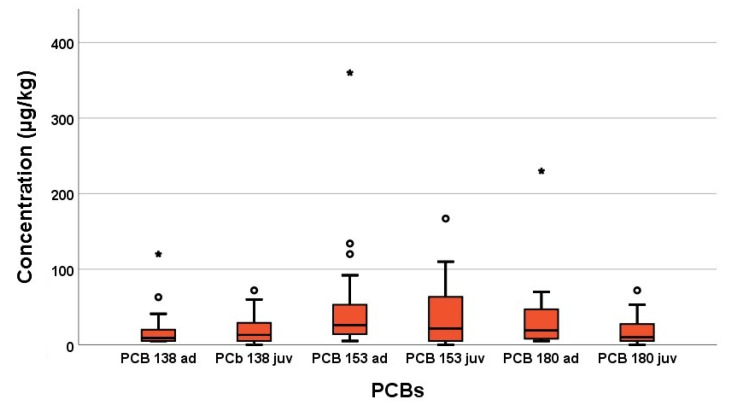
Concentrations of polychlorinated biphenyls in organs of common swifts detected in 2016–2018 in µg/kg wet weight. The box plot shows median (horizontal line in centre), the 25th percentile (box border at bottom) and the 75th percentile (box border at top). The upper and lower ends of the whisker show the maximum and minimum values. The single data points are outliers. The asterisks are extreme outliers.

Furthermore, 4 PCBs were found in the pooled samples of adult and juvenile com-mon swifts. The results are shown in Table 2, Table 3 and Figure 2. There were no significant differences between the PCB concentrations of adult and juvenile swifts. PCB138, PCB153, and PCB180 were found in 100% of adult birds and 85% of juvenile birds, whereas PCB101 was only found in one sample of the juvenile swifts in this study.

## 4. Discussion

The presented data reveal different residues of organochlorine pesticides and polychlorinated biphenyls in the pooled organs of common swifts in the area of Hannover, the swifts’ breeding site. The results of this study show the persistence of long-ago-banned pesticides and their metabolites in birds specialised in the hunting of aerial plankton [26].

The 4,4′-DDE concentrations of adult and juvenile birds in 2018 were markedly risen, although the population of the common swift in Germany had no significant change from 2014 to 2018 [22] and no updated reports of pesticides in migratory birds during these periods. The cause of the increase remains questionable due to the limited availability of specific literatures of this migratory bird.

The mean concentration of residues in swifts from the area in Hannover are lower than the averages of the previously investigated swifts from Rome, Italy, when compared to the lowest mean concentration of their organ samples [23]. Unlike the Italian study, DDT was not detected in any of our specimens. Only its prominent metabolite, 4,4′-DDE is presented. Thus, it is assumable that our birds were exposed to 4,4′-DDE from the historical application of parent DDT, or the exposed DDT residues were already metabolised to 4,4′-DDE in birds in four months prior to the investigation [27]. The body elimination of 4,4′-DDE is relatively slower than other pesticides—its half-life is 229 and 250 days in the studies of grackles [28] and pigeons [29], respectively. Other bird families with different dietary habits show higher contamination levels, for example, predatory birds in Spain [30] were found with higher concentration in liver; which liver sample likely represents the median concentration between fat and muscle samples in regular pesticide-fed birds [27]. Seabird species in Chile also possessed higher pesticide concentrations than the swifts in our study [31]; when referred to the ratio in experimental studies that pesticide residues could be around three times higher in bird’s tissues than in its eggs [27,32]. For the birds with similar dietary habits, the detected 4,4′-DDE levels of insectivorous passerines (carcass concentration) in North America are close to our results [33]. To our knowledge, there is no specific study that shows how common swift are affected by pesticides. Although the average of detected pesticide concentrations is far lower than the available data known to cause fatal effects in birds [11], pesticides cause neurological disorders in birds and endocrine disruption through their influence on the thyroid and steroid hormones. These endocrine disruptions have been considered to be the reason for the lower breeding performance in birds [6,34,35]. An experiment in barn owls shows that the accumulation of residues is more likely to be found in bodies than in eggs [32], which is even more obvious in the feeding experiment of laying hens [27]. Thus, the 4,4′-DDE concentrations in our investigations (0–216 μg/kg ww, in pooled organs) were not high enough to cause eggshell thinning. The record for the lowest concentration that caused eggshell thinning was found in the golden eagle (*Aquila chrysaetos*), which had at least 100 μg/kg ww of DDE residue in eggs. These determined concentrations would reduce the eggshell thickness by around 3% [36], based on the Radcliffe thickness index [37]. However, the higher percentage of eggshell thickness loss may not impact hatchability or juvenile development of some bird species, e.g., the common kestrel [10].

The detected concentrations of PCB in our investigations ranged from 0 to 360 μg/kg ww, which seem to be relatively low [11]. However, the adverse effects of PCB contamination on birds may depend on the susceptibility of the species. It can cause their behavioural and reproductive changes in other bird species, for example, the reduced nest site attentiveness of the glaucous gull (*Larus hyperboreus*) when the PCBs are above 50 μg/kg ww in the blood [38], and at 200 μg/kg ww in the egg; it will extend the incubation time of the zebra finch (*Taeniopygia guttata*) [39]. However, the result of pesticide concentrations found in this study may have been underestimated in an individual bird, because each sample was analysed from the pooled organs of two common swifts. As the physically healthy common swifts were not included in this study at all, it could also lead to a biased result since the samples were only taken from injured birds, and the sample numbers are limited to conclude for the whole healthy population. At this moment, we could not identify that pesticides are the underlying cause of the accident of common swifts brought to the clinic.

It can be assumed that the food of the swifts, aerial insects, is the main source of contamination because these birds can only catch their prey in the air [21,40,41]. Residues in commercial edible insects for human consumption were studied previously [42]. As far as we could find, no similar study of sufficient samples within related locations has yet been conducted with prey insects of insectivorous birds. The transmission of chemical residues to insects is possible during the arthropod lifecycle, which is associated with the ground soil and sediment [43]. From the covered study area, the monitoring of ground residues in 1994–2004 showed that DDTs (DDT, DDE and DDD), PCBs (28, 52, 101, 138, 153 and 180), HCB and lindane could be found in 80 to 100% of samples from the soil observation areas in the forest of Lower Saxony [44,45]. Moreover, OCPs and PCBs were detected in fish from the rivers (Aller, Elbe, Ems, Oste and Weser) in Lower Saxony. DDT and its breakdown products were found in 98.8%, followed by HCB in 85.8%, dieldrin in 27.8% and lindane in 24.3% of the fish samples in these rivers. In addition, PCBs were found in 100% of the PCB-analysed fish samples [46]. This indicates that the water is contaminated by environmental wastes washed down into the ground and ultimately into rivers [17]. The intestinal parasites of swifts, trematodes and cestodes, are likely derived from emergent aquatic insects from the ecosystems where the swifts hunt for their prey [47]. Besides organochlorines, mercury was also found in common swifts in the area of Hannover, Germany [48]. Mercury is one of the heavy metals of global concern that have been found in the aquatic ecosystem over many decades [49]. Those common swifts may expose to mercury through consuming emergent aquatic insects and contaminated arachnids [50].

The significant differences between the 4,4′-DDE concentrations of adult and juvenile swifts can be explained by long-term storage in adults and/or a higher environmental impact in the wintering areas in Africa, where DDT is still used for malaria prophylaxis [14,15]. The young common swifts have less chance of exposure to residues than the adults, who may be exposed to contaminants during the over six months of migration on their travel routes, stopover sites and wintering ground in Africa [20,51]. Adult common swifts are documented to spend their breeding time in the urban areas of Europe for around 15.6 weeks, whereas the juveniles leave these after 6 weeks for Africa [52]. Some of the organochlorines may also have been transferred from the adult swifts to the juveniles. A feeding experiment in chickens demonstrated DDT transfer from DDT-fed hens to their chicks [53].

The results of this study can be applied to other bird species with similar diets, for example, swallows. Moreover, other pesticides were not discussed in this study, which should also be considered for further evaluation.

## 5. Conclusions

In the present study, different organochlorine pesticide and polychlorinated biphenyl residues (4,4′-DDE, dieldrin, HCB, lindane, PCB101, PCB138, PCB153, and PCB180) were found in common swifts, although the use of these compounds was prohibited in Europe many years ago. Adult swifts have significantly higher levels of 4,4′-DDE and dieldrin than juveniles, which could be partially explained by their having more chance of exposure to more polluted areas during their long-distance migration, e.g., travel routes, stopover sites and wintering in Africa, while juvenile common swifts displayed persistent residues taken conceivably from the urban area where their nests are located. However, the average of detected concentrations in our study was far lower than those available data known to cause fatal effects in birds [11].

## Figures and Tables

**Table 1:** The quantification limits of the analysed organochlorine pesticides (OCPs) and polychlorinated biphenyls (PCBs) in the used detection method, GC-TOF/MS (ASU § 64 LFBG/DFG S19).

List of Analysed Chemicals	Quantification Limits (mg/kg)
2,4′-DDE	0.005
2,4′-DDT	0.005
2,4′-DDD	0.005
4,4′-DDE	0.005
4,4′-DDT	0.005
4,4′-DDD	0.005
Alachlor	0.010
Aldrin	0.005
Alpha-Endosulfan	0.005
Beta-Endosulfan	0.005
Beta-Hexachlorocyclohexane	0.005
Bromophos-ethyl	0.010
Bromophos-methyl	0.010
Bromopropylate	0.010
Buprofezin	0.010
Chlorobenzilate	0.010
Chlorfenson	0.010
Chloroneb	0.010
Chlorpyrifos-ethyl	0.010
Chlorpyrifos-methyl	0.010
Chlorthal-dimethyl	0.010
Chlorothalonil	0.010
Chlorthion	0.010
Delta-Hexachlorocyclohexane	0.005
Dicofol	0.010
Dieldrin	0.005
Endosulfan sulfate	0.005
Endrin	0.005
Epsilon-Hexachlorocyclohexane	0.010
Fenson	0.010
Gamma-Chlordane	0.005
Heptachlor	0.005
Heptachlorepoxide-cris	0.005
Heptachlorepoxide-trans	0.005
Hexachlorobenzene (HCB)	0.005
Isodrin	0.005
Jodfenphos	0.010
Lindane (Gamma-Hexachlorocyclohexane)	0.005
Methoxychlor	0.005
Mirex	0.005
Nitrofen	0.010
Parathion-ethyl	0.010
Parathion-methyl	0.010
Pentachloroaniline	0.010
PCB IUPAC-Nr.28	0.005
PCB IUPAC-Nr.52	0.005
PCB IUPAC-Nr.101	0.005
PCB IUPAC-Nr.138	0.005
PCB IUPAC-Nr.153	0.005
PCB IUPAC-Nr.180	0.005

**Table 2:** Concentrations of organochlorine pesticides and polychlorinated biphenyls in pooled organ samples of adult and juvenile common swifts detected in each year from 2016 to 2018 in μg/kg wet weight (ww): Mean ± SD ^a^ (Median; Xmin ^b^–Xmax ^c^).

Compounds	2016		2017		2018	
	Adult Swifts (*n* ^d^ = 5)	Juvenile Swifts (*n* ^d^ = 5)	Adult Swifts (*n* ^d^ = 5)	Juvenile Swifts (*n* ^d^ = 8)	Adult Swifts (*n* ^d^ = 11)	Juvenile Swifts (*n* ^d^ = 7)
4,4′-DDE	10 ± 6	6 ± 4	59 ± 85	7 ± 6	126 ± 41	34 ± 64
	(10; 5–20)	(5; 0–10)	(5; 5–200)	(5; 0–20)	(124; 60–216)	(14; 0–179)
	5/5	4/5	5/5	7/8	11/11	6/7
Dieldrin			2 ± 4.5	0.6 ± 2	3 ± 3	
			(0; 0–10)	(0; 0–5)	(5; 0–5)	
	0/5	0/5	1/5	1/8	6/11	0/7
HCB	2 ± 4	2 ± 4	10 ± 12	3 ± 3	5 ± 1.5	5 ± 0
	(0; 0–10)	(0; 0–10)	(5; 0–30)	(5; 0–5)	(5; 5–10)	(5; 5–5)
	1/5	1/5	4/5	5/8	11/11	7/7
Lindane		1 ± 2	1 ± 2	1 ± 2	3 ± 2.5	
		(0; 0–5)	(0; 0–5)	(0; 0–5)	(5; 0–5)	
	0/5	1/5	1/5	2/8	7/11	0/7
PCB138	5 ± 0	10 ± 17	37 ± 48	28 ± 28	18 ± 18	17 ± 7
	(5; 5–5)	(5; 0–40)	(25; 5–120)	(20; 0–72)	(11; 5–63)	(16; 9–28)
	5/5	3/5	5/5	7/8	11/11	7/7
PCB153	6 ± 2	4 ± 4	118 ± 141	60 ± 59	47 ± 36	32 ± 19
	(5; 5–10)	(5; 0–10)	(62; 24–360)	(42; 0–167)	(39; 14–134)	(23; 14–67)
	5/5	3/5	5/5	7/8	11/11	7/7
PCB180	5 ± 0	10 ± 17	74 ± 90	25 ± 25	31 ± 17	17 ± 17
	(5; 5–5)	(5; 0–40)	(47; 10–230)	(15; 0–72)	(28; 8–55)	(11; 5–53)
	5/5	3/5	5/5	7/8	11/11	7/7
PCB101				10		
	0/5	0/5	0/5	1/8	0/11	0/7

^a^ Standard deviation. ^b^ Minimum concentration. ^c^ Maximum concentration. ^d^ Paired samples.

**Table 3:** Different concentrations of organochlorine pesticides and polychlorinated biphenyls between pooled organ samples of adult and juvenile common swifts detected in 2016–2018 in μg/kg ww: Mean ± SD ^a^ (Median; Xmin ^b^–Xmax ^c^).

Compounds	Adult Swifts (*n* ^d^ = 21)	Juvenile Swifts (*n* ^d^ = 20)	*p* ^e^
4,4′-DDE	83 ± 70 (97; 5–216)	17 ± 39 (5; 0–179)	0.002
Dieldrin	2 ± 3 (0; 0–10)	0.3 ± 1 (0; 0–5)	0.023
HCB	6 ± 6 (5; 0–30)	3 ± 2 (5; 0–10)	0.086
Lindane	2 ± 3 (0; 0–5)	0.8 ± 2 (0; 0–5)	0.099
PCB138	20 ± 30 (9; 5–120)	20 ± 20 (13; 0–70)	0.771
PCB153	50 ± 80 (30; 5–360)	40 ± 50 (20; 0–170)	0.417
PCB180	35 ± 50 (19; 5–230)	18 ± 20 (10; 0–72)	0.128
PCB101	-	10	-

^a^ Standard deviation. ^b^ Minimum concentration. ^c^ Maximum concentration. ^d^ Paired samples. ^e^ Asymptomatic significance (2-tailed), *p*-value.

## Data Availability

The data presented in this study we included in the article here.
